# Prevalence and quantity of lymph nodes at mediastinal stations in patients with lung cancer: lessons from 181 bilateral mediastinal lymphadenectomies

**DOI:** 10.1186/s13019-024-02928-z

**Published:** 2024-07-02

**Authors:** Łukasz Trybalski, Jakub Szadurski, Lanjun Zhang, Jarosław Kużdżał, Akif Turna, Wei-dong Wang, Janusz Warmus, Gizem Ozcibik Isık, Katarzyna Żanowska, Piotr Kocoń

**Affiliations:** 1 Department of Thoracic Surgery, John Paul II Hospital, Cracow, Poland; 2 Department of Thoracic Surgery, Sun Yat-Sen University, Guangzhou, China; 3 Department of Thoracic Surgery, Jagiellonian University Collegium Medicum, John Paul II Hospital, ul. Prądnicka 80, Cracow, 310202 Poland; 4 Department of Thoracic Surgery, Cerrahpasa Medical School, Istanbul University-Cerrahpasa, Istanbul, Turkey

**Keywords:** Lung cancer, Anatomy, Lymph nodes, Mediastinum

## Abstract

**Background:**

This study evaluated the prevalence and quantity of lymph nodes at particular stations of the mediastinum in patients with lung cancer. These data are important to radiologists, pathologists, and thoracic surgeons because they can serve as a benchmark when assessing the completeness of lymph node dissection. However, relevant data in the literature are scarce.

**Methods:**

Data regarding the number of lymph nodes derived from two randomised trials of bilateral mediastinal lymph node dissection, the BML-1 and BML-2 study, were included in this analysis. Detectable nodes at particular stations of the mediastinum and the number of nodes at these stations were analysed.

**Results:**

The mean number of removed nodes was 28.67 (range, 4–88). Detectable lymph nodes were present at stations 2R, 4R, and 7 in 93%, 98%, and 99% of patients, respectively. Nodes were rarely present at stations 9 L (33%), and 3 (35%). The largest number of nodes was observed at stations 7 and 4R (mean, 5 nodes).

**Conclusion:**

The number of mediastinal lymph nodes in patients with lung cancer may be greater than that in healthy individuals. Lymph nodes were observed at stations 2R, 4R, and 7 in more than 90% of patients with lung cancer. The largest number of nodes was observed at stations 4R and 7. Detectable nodes were rarely observed at stations 3 and 9 L.

**Trial registration:**

ISRCTN 86,637,908.

## Background

The prevalence and number of detectable nodes at particular stations of the mediastinum are important to radiologists, pathologists, and surgeons who perform mediastinal lymphadenectomy and mediastinoscopy. Unfortunately, the number of lymph nodes in patients with non-small cell lung cancer (NSCLC) has not been specifically studied, and relevant data are scarce. Most studies of the anatomy of mediastinal lymphatics have been based on investigations of groups without cancer and analysed radiological or autopsy findings rather than surgical specimens [1–4]. Bilateral mediastinal lymphadenectomy (BML) is not routinely performed for patients with lung cancer; however, it provides an opportunity to study the anatomy of mediastinal nodal compartments in surgical practice.

## Materials and methods

This observational study aimed to determine the prevalence (defined as the percentage of patients with any detectable lymph nodes present in particular mediastinal stations) and quantity of lymph nodes at mediastinal stations in patients with lung cancer by evaluating data retrieved from two prospective, randomised trials, the BML-1 study [5] and the BML-2 study (ISRCTN 86,637,908), that assessed the value of BML for the surgical treatment of NSCLC. The protocols of the BML-1 and BML-2 study have been approved by the Bioethical Committee of the Jagiellonian University (K/ZDS/002337 and 1072.6120.91.2017), where the full protocol of the study is available. Both, BML-1 and BML-2 studies have been performed in accordance with the Declaration of Helsinki.

The sample size was not determined before this study was performed because of its design. The anatomy of the mediastinal lymphatics of a group of patients who underwent surgery at the Department of Thoracic Surgery, Jagiellonian University Medical College, John Paul II Hospital, Cracow, Poland, the Department of Thoracic Surgery, Sun Yat-Sen University, Guangzhou, China, and the Department of Thoracic Surgery, Cerrahpasa Medical School, Istanbul University-Cerrahpasa, Istanbul, Turkey, were evaluated. The inclusion criteria for the BML-1 study and BML-2 study were as follows: adult patients with confirmed or suspected NSCLC stages I to IIIB; preoperative staging was routinely performed using chest radiography, computed tomography, positron emission tomography-computed tomography, abdominal ultrasonography, bronchoscopy, endobronchial ultrasonography and endoscopic ultrasonography; and patients were suitable candidates for lung resection. The exclusion criteria were as follows: history of malignancies other than non-melanoma skin cancer; use of chemotherapy or chemoradiotherapy; pathological confirmation of tumours other than NSCLC; ground-glass opacity lesions; and lack of informed consent. In both studies patients were randomized in a 1:1 ratio to the group in which systematic (unilateral) lymph node dissection or BML was performed. The technique of BML is summarized below.

Bilateral mediastinal lymph node dissection was performed using both a cervical incision and thoracotomy or video-assisted thoracoscopic surgery (VATS) during one procedure. For right-sided surgery, the procedure began in a supine position with a cervical incision and exploration of stations 2 L, 4 L, and 7. Subsequently, patients were repositioned to the lateral decubitus position and – using the open or VATS approach – appropriate lung resection was performed and lymph node stations 2R, 3, 4R, 7, 8, and 9R were dissected. For left-sided surgery, the procedure began with a cervical incision and exploration of stations 2R, 2 L, 4R, 4 L, and 7. Subsequently, using the open or VATS approach, appropriate lung resection was performed and lymph node stations 5, 6, 7, 8, and 9 L were dissected. The dissection technique has been described in detail elsewhere [5]. During the procedure, the nodes from each station were labelled separately and counted by the operating surgeon to avoid their overestimation. Overestimations could occur if the number is assessed based on the pathology report because some nodes are inevitably removed in pieces. Lymph nodes were classified according to the 8th edition of the TNM staging system for lung cancer [6]. Data regarding the mediastinal lymph nodes of all patients who underwent BML during both studies were pooled for the final analysis. The prevalence of detectable lymph nodes at each mediastinal station was determined, and the node quantities are presented as the mean and range.

## Results

**Fig. 1:**
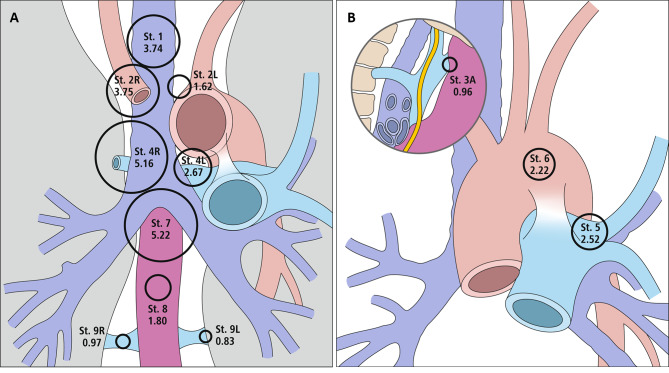
**A**-**B** Mean numbers and range (in brackets) of lymph nodes in particular mediastinal stations

**Fig. 2:**
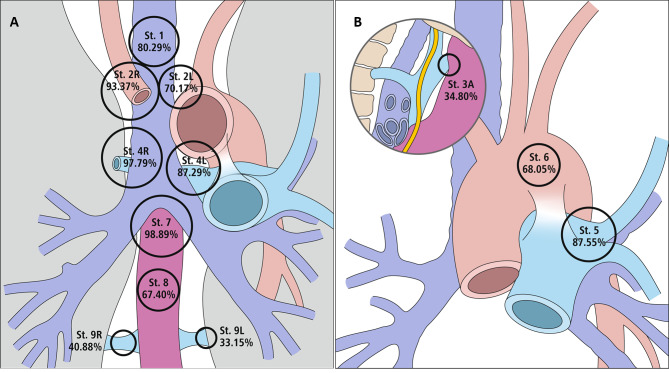
**A**-**B** Prevalence of lymph nodes in particular mediastinal stations

The study group comprised 181 patients who underwent bilateral mediastinal dissection. The characteristics of this group are presented in Table 1. The mean number of removed nodes was 28.67 (range, 4–88). At stations 2R, 4R, and 7, detectable lymph nodes were present in 93%, 98%, and 99% of patients, respectively. Nodes were rarely present at stations 9 L (33%), and 3 (35%). The largest number of nodes was encountered at stations 7 and 4R (mean, 5 nodes). The prevalence and number of mediastinal lymph nodes at each station are listed in Table 2 and presented in Figs. 1 and 2.

**Table 1:** Characteristics of the study group

Variable	
Age, range (median)	24–77 (61)
Sex	
F/M, n F/M, %	71/110 39.2/60.8
Tumour location, n (%)	
RUL RML RLL RCE LUL LLL LCE	57 (31.5) 13 (7.2) 35 (19.3) 3 (1.6) 46 (25.4) 22 (12.2) 5 (2.8)
Histology, n (%)	
ADC SCC ASC LCC other	123 (68) 41 (22.6) 5 (2.8) 2 (1.1) 10 (5.5)
Stage, n (%)	
IA IB IIA IIB IIIA IIIB	75 (41.4) 34 (18.8) 15 (8.3) 22 (12.2) 26 (14.3) 9 (5.0)
Surgical approach	
VATS/RATS Open	24 (13.3) 157 (86.7)
Resection type	
RUL RML RLL UBL LBL RPN CUL LIN LUL LLL LPN	55 (30.4) 13 (7.2) 29 (16.1) 3 (1.7) 8 (4.4) 1 (0.5) 3 (1.7) 1 (0.5) 41 (22.6) 18 (9.9) 9 (5.0)

ADC: adenocarcinoma; ASC: adenosquamous carcinoma; CUL: culmenectomy; F: female; LBL: lower bilobectomy; LCC: large cell carcinoma; LCE: left central excision; LIN: lingulectomy; LLL: left lower lobe/lobectomy; LPN: left pneumonectomy; LUL: left upper lobe/lobectomy; M: male; RATS: robotic-assisted thoracoscopic surgery; RCE: right central excision; RLL: right lower lobe/lobectomy; RML: right middle lobe/lobectomy; RPN: right pneumonectomy; RUL: right upper lobe/lobectomy; SCC: squamous cell carcinoma; UBL: upper bilobectomy; VATS: video-assisted thoracoscopic surgery

**Table 2:** Prevalence and number of mediastinal lymph nodes* according to station in 188 patients with lung cancer

Station	Prevalence %	Mean number (SD)	Range	Median (IQR)
1	80.29	3.74 (3.49)	0–18	3 (4.0)
2R	93.37	3.75 (2.81)	0–15	3 (3.0)
2 L	70.17	1.62 (1.65)	0–9	1 (2.0)
3 A	34.80	0.96 (2.11)	0–16	0 (1.0)
4R	97.79	5.16 (3.29)	0–18	4 (3.0)
4 L	87.29	2.67 (2.16)	0–11	2 (3.0)
5	87.55†	2.52 (1.87)†	0–13†	0 (2.0)
6	68.05†	2.22 (1.82)†	0–11†	0 (1.0)
7	98.89	5.22 (3.60)	0–25	5 (4.0)
8	67.40	1.80 (1.79)	0–11	2 (3.0)
9R	40.88	0.97 (1.42)	0–8	0 (1.0)
9 L	33.15	0.83 (1.49)	0–8	0 (1.0)
Overall	100	28.67	4–88	

*According to the International Association for the Study of Lung Cancer classification [1]

†Because stations 5 and 6 were not dissected during surgery for right-sided tumours, these data were calculated in a group the 72 patients who underwent left-sided surgery. SD – standard deviation; IQR – interquartile range

## Discussion

To the best of our knowledge, this is the first study to evaluate the surgical anatomy of the mediastinal lymph nodes based on the results of bilateral mediastinal dissection performed during lung cancer surgery. These data are important to radiologists, pathologists, and thoracic surgeons because they can serve as a benchmark when assessing the completeness of lymph node dissection. Previous studies involved cadaveric and radiological studies or diagnostic explorations of the mediastinum. Beck and Beattie were the first to report data regarding the number of mediastinal lymph nodes [1]. According to their study involving five cadavers, the average number of lymph nodes was 53; however, the number of autopsies performed was limited, and the authors distinguished only four groups of mediastinal nodes (anterior mediastinum, peribronchial, subcarinal, and paratracheal nodes). Genereux and Howie reported the number of mediastinal lymph nodes without any nodal pathology observed in 39 patients using computed tomography (CT) and during dissection at the time of autopsy in 12 cadavers [2]. These authors divided the nodes into four zones: related to the innominate vein, pretracheal space, pericarinal/subcarinal space, and aortopulmonary window, and concentrated on the node size rather than on the number of nodes. Although they reported some data regarding the number of nodes found using CT (mean, 5.7 lymph nodes), they did not report any data regarding the number of nodes found during autopsy.

Two studies have reported the number of mediastinal lymph nodes classified according to the American Thoracic Society [7]. Glazer et al. reported the CT data of 56 patients and Kiyono et al. reported the data of 40 cadavers obtained during dissection at the time of autopsy [3, 4]. Individuals with any malignancy or chest inflammation were excluded from these studies. Summarised data from these two studies are presented in Table 3.

**Table 3:** Prevalence and number of mediastinal lymph nodes according to station* in patients without intrathoracic pathology in the literature

Nodal station	Glazer et al. [6]			Kiyono et al. [7]		
	Prevalence %	Mean ± SD	Maximum, *n*	Prevalence, %	Mean ± SD	Maximum, *n*
2R	94.6	2.1 ± 1.3	6	80	2.5 ± 2.2	11
2 L	75	1.9 ± 1.6	6	68	2.1 ± 2.2	7
4R	100	3.2 ± 2.0	10	98	4.8 ± 2.8	11
4 L	83.9	2.1 ± 1.6	7	98	4.5 ± 2.9	16
5	58.9	1.2 ± 1.1	3	58	1.1 ± 1.4	6
6	85.7	4.8 ± 3.5	12	85	4.7 ± 3.9	15
7	94.6	1.7 ± 1.1	6	100	2.9 ± 1.4	6
8R	57.1	1.0 ± 1.1	4	58	1.2 ± 1.4	6
8 L	44.6	0.8 ± 1.2	6	50	1.1 ± 1.4	5
9R	ns	ns	ns	10	0.1 ± 0.4	2
9 L	ns	ns	ns	35	0.5 ± 0.8	3
Overall	-	18.8	-	-	25.5	-

*According to the American Thoracic Society classification [5]

ns: not stated; SD: standard deviation

Two other studies have provided data regarding the number of lymph nodes removed during right or left thoracotomy for patients with lung cancer. Although the authors did not distinguish the nodes according to the American Thoracic Society or Mountain-Dresler classifications and used a simplified classification (superior mediastinal, inferior mediastinal, and para-aortic nodes), they reported the number of removed mediastinal lymph nodes. The numbers of nodes on the right and left sides were determined separately (21 ± 13 and 17 ± 10, respectively) [8, 9]. However, their sum should not be directly compared with the results of other authors because station 7 nodes were removed during thoracotomy on each side, and those at station 1 and station 2 L were not removed regardless of the side of the thoracotomy.

According to a retrospective study that assessed the value of transcervical extended mediastinal lymphadenectomy in lung cancer staging, 15 to 85 (mean, 38.9) lymph nodes were removed; however, station 9 was not dissected, and the number of nodes removed from particular mediastinal stations was not reported [10].

Based on these results, three issues should be addressed. First, the sensitivity of the CT-based assessments performed by Genereux and Howie and Glazer et al. [2, 3] was dependent on the CT scanner generation used in 1980s and slice thickness; the risk of missing small nodes is substantial with thick slices. Second, the numbers of detectable mediastinal nodes in individuals without chest inflammation or malignancy may differ from those of patients with NSCLC. The aforementioned studies included populations without malignancy or chest inflammation [3, 4]; it should be emphasized that the numbers of lymph nodes reported by those studies were approximately two-fold less than those reported by other studies involving patients with lung cancer and our team [8, 9]. Third, the simplified classifications used in these studies do not correspond to the present 8th edition of the TNM system.

Additionally, the distribution of nodes differed. For individuals without chest pathology, the largest number of nodes was encountered in both the left paratracheal (stations 2 L and 4 L) and preaortic regions (station 6), and relatively fewer nodes were encountered in the subcarinal region (station 7), suggesting that changes in the mediastinal lymphatic system of patients with lung cancer may lead to inadequate results of anatomical studies in clinical settings. However, the nodal stations with the largest and smallest prevalence of lymph nodes did not differ between patients with lung cancer and healthy individuals.

The strengths of our study include its multicentre design, large number of analysed patients and use of data from real-world surgical practice. Therefore, the applicability of these results to clinical practice may be better than that of the results of purely anatomical studies. Data we present may be used as a reference point for assessment of completeness of lymph node dissection during lung cancer surgery. Also, we used the current TNM classification, instead the simplified divisions used in previously published papers [1, 2, 4, 8]. Stations 5 and 6 were not dissected during right-sided surgery, which was the main limitation of this study. However, because metastases from right lung tumours to these two stations are very rare, the clinical importance of this limitation seems negligible.

## Conclusions

Patients with lung cancer may have more detectable mediastinal lymph nodes than healthy individuals. Lymph nodes were most often observed at stations 2R, 4R, and 7 in patients with lung cancer. The largest number of nodes was observed at stations 4R and 7, while detectable nodes were least often observed at stations 3 and 9 L.

## Data Availability

The datasets used and/or analysed during the current study available from the corresponding author on reasonable request.
